# A History of COVID-19 in Pregnancy: A Narrative Review

**DOI:** 10.3390/jcm12175722

**Published:** 2023-09-01

**Authors:** Shahrukh Chaudhry, Omar Aboudawoud, Ghislain Hardy

**Affiliations:** 1 Paul L. Foster School of Medicine, Texas Tech University Health Sciences Center El Paso, El Paso, TX 79905, USA; shahchau@ttuhsc.edu (S.C.); omabouda@ttuhsc.edu (O.A.); 2 Department of Obstetrics and Gynecology, Texas Tech University Health Sciences Center El Paso, El Paso, TX 79905, USA

**Keywords:** COVID-19, COVID placentitis, pregnancy, maternal health, SARS-CoV-2

## Abstract

Coronavirus disease 2019 (COVID-19) caused by severe acute respiratory syndrome coronavirus 2 (SARS-CoV-2) has rapidly spread across the world causing a global pandemic. During a pandemic, it becomes increasing important to evaluate the effects on specific populations at risk. In this narrative review, we analyzed the literature regarding COVID-19 infection on the pregnant population as they are at increased risk of infection. COVID-19 did seem to significantly increase the risk of obstetric complications, specifically in underserved and marginalized populations. In general, COVID-19 rarely directly infected the fetus and placenta, apart from a very rare complication called COVID placentitis. In actuality, the mothers were at greatest direct risk due to COVID-19 infection. The most important takeaway from this pandemic is the prospective lesson and effect it had on social determinants of health. Women did not have safe access to antenatal care, leading to a plethora of indirect obstetric complications due to COVID-19. In conclusion, it was women who suffered from the pandemic, not the placenta nor the fetus. It is our duty as physicians to protect pregnant women, allowing the placenta to protect the fetus.

## 1. Introduction

In December 2019, four cases of pneumonia of unknown etiology were reported in Wuhan, China to the World Health Organization (WHO) [1]. Since then, coronavirus disease 2019 (COVID-19), found to be caused by severe acute respiratory syndrome coronavirus 2 (SARS-CoV-2), has rapidly spread across the globe. On 12 March 2020, COVID-19 was officially declared a global pandemic by the World Health Organization (WHO) [2]. The COVID-19 pandemic continued to evolve with the emergence of several new variants until 11 May 2023, when the WHO declared the end of the COVID-19 public health emergency [2]. Many scholars feel that, because of vaccination and natural immunity, we have now transitioned from pandemic COVID-19 to endemic COVID-19. There are still new variants being described all the time and our community will need to remain vigilant including monitoring the effect on mothers and babies. Even after clearing the initial infection, people can suffer from Long COVID, a debilitating illness that occurs in greater than 10% of COVID-19 infections. At least 65 million cases of Long COVID have been reported worldwide, with the number increasing daily [3]. Studies are needed to evaluate the impact of Long COVID on pregnancy.

As the pandemic spread across the world, it became increasingly important to observe and analyze the effect of SARS-CoV-2 infection on populations known to be at increased risk of infection including pregnant patients. During the beginning of the pandemic, there were very limited data regarding the effects of SARS-CoV-2 on the pregnant patient and fetus. A number of studies thus began surfacing attempting to measure the effects that COVID-19 infection has on maternal health, fetal health, the placenta, and pregnancy outcome. 

According to the literature, the majority of pregnant patients and babies infected with SARS-CoV-2 have only suffered a mild illness and overall have done well [4]. However, there were reported increases in several pregnancy complications. For example, several studies have shown that COVID-19 infection during pregnancy increases the risk for both preeclampsia and preterm birth. The severe form of COVID-19 disease (defined as a respiratory rate of ≥30 breaths per minute and an oxygen saturation of 93% or less on room air) was strongly associated with gestational diabetes, pre-eclampsia, preterm birth, cesarian delivery, low birth weight and higher rates of admission to the neonatal intensive care unit more than the mild form of disease (defined as positive testing for COVID-19 infection but without severe symptoms) [5]. Mild disease seemed to primarily affect maternal health, with the majority of neonatal outcomes being reassuring [6]. One of the most severe but rare complications secondary to COVID-19 infection was termed COVID placentitis or SARS-CoV-2 placentitis. COVID placentitis can affect > 75% of the placenta by causing diffuse placental parenchymal destruction. This destruction can render the placenta incapable of oxygenating the fetus, leading to placental insufficiency and malperfusion, with the most feared complication being stillbirth or neonatal death [7]. 

In this review, selective examples of the literature published on COVID-19 infection and pregnancy since the emergence of the global pandemic were analyzed. In doing so, we hope to uncover the general overall trends presented by the corresponding literature and extract meaningful lessons to be learned. Thus, we hope to reexamine, with the clarity of hindsight, the biopsychosocial reality of pregnant women during the hazy nightmare we were lucky to survive.

## 2. Methods

The available literature regarding COVID-19 was reviewed using electronic databases such as MEDLINE/PubMed, Google Scholar, and Scopus. Publications from the last 4 years (from 2019 to 2023) were analyzed. Our search was limited to relevant articles written in the English language. The key terms searched included “COVID-19”, “SARS-CoV-2”, “COVID in pregnancy”, “COVID placentitis”, and “COVID effect on fetal and maternal health”. Our search focused on review articles, systematic reviews, meta-analysis studies and case reports. As COVID-19 is still an emerging topic in the literature, our search was limited. 

This is not a systematic review of the medicine or science of COVID-19; it is a narrative review with a historical bent that uses a multiperspectival lens and has an underlying philosophical and ethical impetus. The style may be slightly unfamiliar to the usual reader of medical literature but the pandemic was a special circumstance and thus calls for a special response. Old conventions lack the vocabulary to fully address new unknown collective trauma. We must ask new questions, find new words. In this paper, we seek to enact the calling to find a new voice.

## 3. The Effect of COVID-19 on the Placenta

### 3.1. The Placenta as a Barrier to Viral Infection

It is well known that the human placenta plays an essential role in the modulation of immune responses seen with several viral infections [8]. The placenta has the unique ability to prevent and limit expansion of a viruses’ subsequent transmission to the fetus. In most cases of COVID-19 infection, the fetuses were uninfected and unharmed without any evidence of damage to the placenta, so we assume the placenta served as an effective barrier to fetal infection. Additionally, the placenta was usually successful in eliminating and preventing transmission of the pathogen while remaining largely undamaged itself.

### 3.2. COVID Placentitis Review

**Figure 1:**
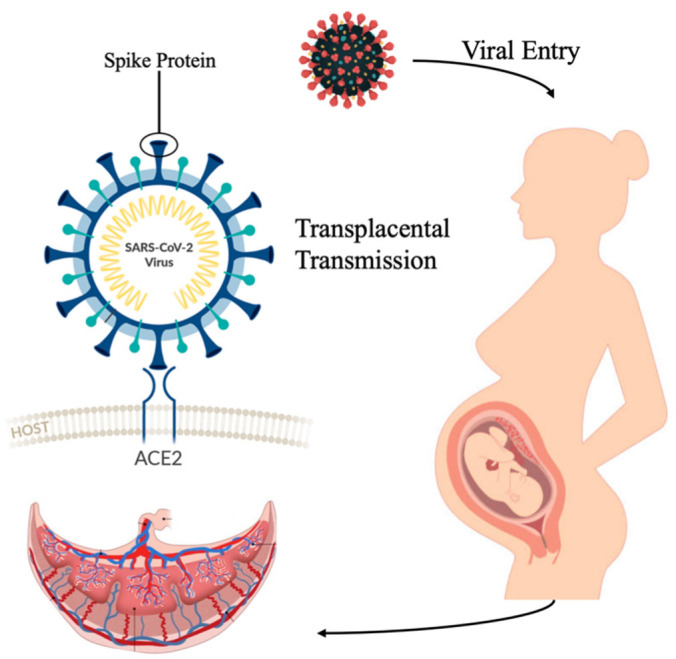
Transplacental transmission of SARS-CoV-2 via the ACE2 receptor.

COVID placentitis is a very rare but severe complication of COVID-19 infection during pregnancy. Its emergence occurred during the Alpha era, which first appeared in November 2020, with a surge in December. Severe complications peaked during the Delta era in late 2020, and there have been no reports in the literature since the beginning of the Omicron era [9,10,11,12]. Many studies focused on evaluating COVID-19 entry factors that allow it to directly attack the placenta. The most widely accepted mechanism currently for SARS-CoV-2 entering host cells is via the angiotensin-converting enzyme 2 (ACE2) receptor [13], the expression of which plays a primary role in COVID-19 infection of the placenta [14] as shown in Figure 1. Based on these observations, SARS-CoV-2, in rare cases, had the capacity to enter the syncytiotrophoblast layer, the boundary between maternal and fetal compartments. The most common lab techniques used to detect the virus in placental tissue included in situ hybridization (ISH) and immunohistochemistry with antibodies against viral spike proteins or nucleocapsid [15]. The placental damage in COVID-19 placentitis was a rapid process, as nearly all reported infections were followed by delivery of stillbirth within approximately 2 weeks or less of the initial diagnosis. The high rate of stillbirth in COVID placentitis is due to asphyxiation secondary to the placental damage, not due to direct fetal infection by the virus [16]. 

In December 2021, Members of The National Institutes of Health/Eunice Kennedy Shriver National Institute of Child Health and Human Development met with the goal of proposing a working definition of placental infection with SARS-CoV-2 during pregnancy. This meeting led to the first standardized definition of what is now known as COVID placentitis. A graded classification regarding the likelihood of placental infection was proposed ranging from definitive, probable, possible, and unlikely. According to their guidelines: definite placental infection with SARS-CoV-2 must show evidence of active replicating virus within placental tissue. Additionally, it was agreed upon, that placental infection be defined using techniques such as in situ hybridization with antisense or sense probes, immunohistochemistry, or rtPCR as these methods allow for virus localization in placental tissue. These experts also stated that future manuscripts reporting COVID placentitis must describe the sampling method and detection technique used in order to avoid misclassification. Finally, recommendations regarding the handling of the placenta, sampling and the use of validated reagents and sampling protocols were also established [15]. 

With regard to the placental pathology in COVID placentitis, macroscopically the placentas may show pale nodules and streaks with involvement of a significant proportion of the placental volume [17]. Histologically, there is a tetrad of findings which includes syncytial necrosis, fibrinoid deposition, intervillous histiocytes and karyorrhectic debris; each of these elements may occur to varying extent in a given case but a significant proportion of the placental territory is involved [15,16,17,18]. These effects are due to the cytopathic (necrotizing) effect of virus on the tissue and the ensuing inflammatory response. The histological differential diagnosis includes Chronic Histiocytic Intervillositis (CHI), Massive Perivillous Fibrinoid (MPFD), and vascular infarcts. Ancillary conformation of the virus by RNA in situ hybridization or immunochemistry helps establish the diagnosis [15].

### 3.3. Brief Overview of Non-Specific Placental Lesions

Outside of the rare context of COVID placentitis, in general overview, in most COVID-19 pregnancies, there was no evidence of viral infection of the placenta nor tissue damage due to virus. However, there is some possible evidence of a general mild increase in non-specific lesions in mothers with COVID-19 which may be attributable to pre-placental maternal unwellness which can impact factors such as placental perfusion and blood coagulation [19]. Several studies reported increased placental pathology following COVID-19 infection including fetal vascular malperfusion (FVM) (35%) and maternal vascular malperfusion (NVM) (46%) while inflammatory findings have rarely been reported [19,20]. These features while possibly mildly increased in COVID-19 are not specific and are common placental pathology lesions. 

Another noteworthy finding reported by Radan et al. is decreased placental weight and alteration of metabolic scaling observed after SARS-CoV-2 infection during pregnancy. This prospective multicenter study included 153 placentas from COVID-19 positive mothers and the main finding was an increased incidence of low placental weight after COVID-19 infection during pregnancy. The study found that 42.5% of placentas had weights below or equal to the 10th percentile, and 19% of placentas had weights below the 3rd percentile [21].

## 4. The Effect of COVID-19 on the Fetus

### 4.1. Vertical Transmission

Vertical transmission (VT) occurs through the transplacental transfer of pathogens or microorganisms during the pregnancy period, during delivery via contact with vaginal secretions or blood, and is even possible via breast milk [22]. VT is confirmed when a PCR test of either umbilical cord blood, amniotic fluid, or neonatal blood is positive within the first 12 h after birth [23]. Multiple meta-analyses have been published regarding VT of COVID-19 with results estimating a range of 3–8% [23]. The criteria for VT in most of these meta-analyses was defined as a positive PCR test for SARS-CoV-2 mRNA within 48 h after birth, not 12 h. This delay in detection allows for transmission of SARS-CoV-2 during delivery and thus a significant proportion of the VT cases may be intrapartum and not transplacental [24]. A potential problem is that many studies could not distinguish VT from transmission occurring in the hospital [22]. According to a more recent classification system, perinatal SARS-CoV-2 transmission is considered proven only if the virus is detected in amniotic fluid collected prior to the rupture of membranes or in neonatal blood drawn early in life. There were several case reports suggesting that COVID placentitis may be a risk factor for VT (refs listed below) but this was never confirmed by a controlled study [25,26,27].

### 4.2. Uninfected but Not Unaffected

The current literature suggests that maternal COVID-19 infection in pregnancy can potentially have a negative impact on the developing fetus, but the extent of this impact is not fully understood. Various studies have reported different outcomes ranging from no adverse overall effects to serious fetal complications such as increased preterm birth, fetal distress and even stillbirth. A systematic review found that pregnant women with COVID-19 were at higher risk of preterm birth, stillbirth, and neonatal death although the overall number of neonatal deaths observed was only 16 in the COVID-19 groups [6]. Di Toro et al. performed a larger and more recent meta-analysis which involved 1100 patients from 24 different studies and concluded that COVID-19 does not have a significant impact on fetal health based on the following; only 1 of 225 newborns exhibited an APGAR score < 7 at 1 min, there were no reports of confirmed VT, and only one newborn required non-invasive ventilation, with the number of symptomatic infants also being very miniscule [28]. In their study, there were a total of three stillbirths and three neonatal deaths, but no clear correlation with infection was reported [28]. A recent meta-analysis published in 2023 by Smith et al. based on 18 studies also failed to establish a link between SARS-CoV-2 infection during pregnancy and fetal growth restriction or still birth [29]. The results of this analysis suggest that fetal and neonatal mortality secondary to maternal COVID-19 infection is low. Thus, there are significant disparities in the literature which can be attributed to a multitude of factors including study design, era/strain that the study included, population/host factors and maternal and neonatal care factors. 

The Developmental Origins of Health and Disease (DOHaD hypothesis initiated by David Barker based on epidemiology tells us that maternal illness, stress and privation can have long-term impacts on the health of the individual, in adulthood, even if they seem well at birth and in childhood. From a mechanistic basis, epigenetic modifications such as non-coding miRNAs, DNA methylation and histone modification are thought to be involved in mediating the effect the early life environment has on future health [30]. This concept can be applied to SARS-CoV-2 infection. COVID-19 causing maternal hypoxia could represent a mechanism for disruption of the fetal and perinatal environment alike. This disruption could alter fetal programming regarding the expression of Angiotensin-converting enzyme 2 (ACE2) and multiple components of fetal organodevelopment as well as at the level of the placenta [31]. The ultimate question is, will there be a Developmental Origins of Health and Disease (DOHaD) impact on the COVID-19 birth cohort due to maternal stress? Only time will tell. Therefore, the effects of this new disease and the pandemic it caused must be analyzed thoroughly in the years to come. Thus, it is important to realize that just because a neonate evades VT they can still be impacted indirectly by increased maternal obstetric complications such as prematurity or maternal respiratory hypoxia, leading to fetal distress and by subtle long-term developmental consequences of gestation and parturition occurring under conditions of maternal unwellness and stress. The latter is difficult to study and what one finds is in part an artifact of how one looks.

## 5. The Effect of COVID-19 on the Mother

Pregnancy places women in a particularly vulnerable state of health requiring special physical and mental care. Therefore, it is important to answer the question of whether pregnant women are indeed at greater susceptibility to COVID-19 infection or have worse disease outcomes when compared to their non-pregnant counterparts. The direct effects of infection aside, maternal healthcare may also be disproportionately affected by a global pandemic due to its negative impact on the world’s healthcare infrastructure and society as a whole. 

### 5.1. Direct Effects

The pregnant state leads to profound physiological effects and immunological alterations in mothers to protect and support the developing fetus. These changes lead to pregnant patients being at an increased risk of infection in general. Congruently, pregnant patients may be at a heightened risk of COVID-19 infection as well [32]. Early studies during the COVID-19 pandemic generally suggested that pregnant individuals suffering from COVID-19 infection did not display increased disease severity when compared to their non-pregnant counterparts. Most cases were reported as either entirely asymptomatic or mildly symptomatic [33]. Common non-specific laboratory findings included leukopenia, lymphopenia, thrombocytopenia and elevated levels of CRP and transaminases with some reports of an increased D-Dimer level [34,35]. Computed tomography (CT) scans of the lungs of pregnant individuals suffering from COVID-19 infection also revealed abnormal findings, often times showing diffuse ground-glass opacities [36]. However, the clinical significance of the laboratory and imaging findings could not be directly correlated to COVID-19 infection alone. More recent studies have seemed to contradict the early reported benign nature of the disease. For example, a large meta-analysis of 400,000 women with symptomatic COVID-19 disease in the United States was published in October 2020 showing that pregnant women were more like to require mechanical ventilation and experience ICU admission and even death [37]. Similarly, a multicenter retrospective case control study performed in Philadelphia on November 2020 found that pregnant women admitted for severe COVID-19 disease were more likely to be intubated, admitted to the intensive care unit, and at an increased risk of morbidity when compared to non-pregnant women with the same disease [38]. Therefore, during the COVID-19 pandemic, particularly with the emergence of more virulent strains, the initial optimism regarding a benign course of COVID-19 in pregnancy yielded to the realization that the disease’s impact was more severe among pregnant individuals.

### 5.2. Indirect Effects

The importance of evaluating the indirect effects of COVD-19 must not be understated. As the pandemic progressed the significant adverse effect on maternal health and healthcare due to the indirect effects of COVID-19 were revealed [39]. In 2020, the United Nation Policy Brief regarding the impact of COVID-19 on women commented that “the pandemic is deepening pre-existing inequalities, exposing vulnerabilities in social, political and economic systems which are in turn amplifying the impacts of the pandemic…with women being disproportionately affected” [40]. Pandemics cause a shift in healthcare resources to focus on the management of patients suffering from the acute infection at hand. This shift can lead to a lack of necessary healthcare for vulnerable patient populations, leading to an increased number of adverse consequences [39]. 

The negative impact on mental health is one of the most overlooked aspects of the COVID-19 pandemic’s indirect effect on maternal health. Pregnant women at baseline are at an increased risk of experiencing mental health issues and the COVID-19 pandemic served to compound this effect [34]. Women were noted to experience increased feelings of depression and anxiety associated with limited access to antenatal care, lack of social support secondary to the social distancing measures placed on them and fear of vertically transmitting the virus to their fetus [41]. Koenan and colleagues performed a global survey of pregnant and postpartum women. They found that over 70% of women reported clinically significant feelings of depression or anxiety and over 40% of women were diagnosed with post-traumatic stress disorder (PTSD) [42]. 

The COVID-19 pandemic also lead to significant reduction in accessing antenatal and postnatal care due to the fear transmitting the virus during clinic visits. In fact, many health services thought to be “non-essential” were postponed [33]. An online survey of 4451 pregnant women in the United States found that one-third of those surveyed reported elevated stress levels secondary to the alterations in their prenatal appointments [33]. Semaan et al. performed a global cross-sectional study regarding antenatal care services and found that clinic hours, in-person visits and number of visitors allowed decreased significantly during the COVID-19 pandemic [43]. Although telemedicine was available to provide antenatal care to some women [33], it was not enough to fill the gap in healthcare access created by the pandemic. 

COVID-19 caused a push for worldwide “social-distancing” and quarantining which lead to the closure of many outpatient clinics for prolonged periods of time. The quarantine recommendations made it difficult for women to access necessary maternal and reproductive healthcare services ranging from routine checkups to abortion [33]. The UN Population Fund released an estimate in 2022 that if COVID-19 related disruption were to continue for 6 more months, 47 million women in approximately 114 low-to-middle-income countries (LMIC) would be unable to use contraceptives, leading to an estimated 7 million unintended pregnancies globally [44]. The International Planned Parenthood Federation released a survey which found that by 5 April 2020, 5633 mobile and community-based sexual and reproductive healthcare facilities had closed across 64 different countries [43]. In LMICs, many pregnant patients were denied entry to hospitals and forced to undergo labor in their homes or even on the streets. This phenomena was particularly prevalent in India [45,46]. Many hospitals across the globe placed limitations on the number of visitors allowed and their duration of stay during and after birth in an effort to mitigate viral transmission [33]. However, this too has the potential to negatively impact the quality of maternal healthcare. Studies conducted on pregnant individuals in the United Kingdom and Nepal found a significant increased incidence of neonatal mortality and stillbirth during the COVID-19 pandemic when compared to the pre-pandemic period. However, the patients experiencing stillbirth and infant mortality often did not have symptoms of COVID-19 infection. This suggests the negative outcomes experienced may have been due to the reduction in perinatal care visits and labor management secondary to the reallocation of resources towards COVID-19 patients during the pandemic, not due to the direct effects of viral infection on maternal physiology [47]. The shortcomings of LMICs in their ability to meet the demands of a global pandemic threatened both the mental and physical health of pregnant patients severely, displaying the critical indirect obstetric effects of COVID-19. 

### 5.3. Paradigm Shift of Obstetric Complications

The tenth revision of the International Classification of Diseases (ICD) is used to classify maternal deaths during pregnancy, childbirth, and the postpartum period (ICD-Maternal Mortality [MM]). Maternal deaths are divided into two categories: direct obstetric deaths, which are caused by obstetric complications during pregnancy or childbirth, and indirect obstetric deaths, which result from pre-existing or pregnancy-related diseases that are worsened by the physiological effects of pregnancy [48]. Indirect obstetric complications are more prevalent in developed countries possibly because our advancing technology effectively pushes the limits of natural fertility. With the use of advanced reproductive technologies age of maternal gestation is rising, leading to an increased number of women with pre-existing co-morbidities (i.e., diabetes mellitus, cardiovascular disease) becoming pregnant. Alternatively, direct obstetric causes are more likely to be observed in developing or LMICs due to lack of adequate access to maternal healthcare. A meta-analysis performed by Say et al. found that of the direct causes of maternal death, hemorrhage was the leading cause, followed by sepsis secondary to infection and hypertensive disorders [49] in underdeveloped countries worldwide.

Although the literature does show COVID-19 causing a moderate amount of direct complications in pregnancy, the magnitude of impact of COVID-19 had on pregnancy was largely indirect in nature. COVID-19 has an infectious disease epidemiology and thus mainly affects the marginalized, not the affluent, yet is associated with a high burden of indirect obstetrical complications. Thus, the COVID-19 pandemic effectively reverses the paradigm commonly seen with respect to direct and indirect obstetric complications. 

### 5.4. COVID-19 Vaccination during Pregnancy

The impact of COVID-19 vaccination on pregnancy has been a subject of intense research. The need for vaccination prior to or during pregnancy is vital in protecting the health of the mother and fetus. The Centers for Disease Control and Prevention reports that mRNA COVID-19 vaccines are generally safe and effective during pregnancy in recently updated guideline [50]. The US Vaccine Safety Datalink system performed a case–control study including 31,080 people vaccinated during pregnancy and found no indication that COVID-19 vaccination is linked to stillbirth [51]. The vaccines’ efficacy in preventing severe COVID-19 outcomes has been observed to be significant, reducing the risk of hospitalization and other complications such as stillbirths during pregnancy [50]. Of note, vaccine-generated antibodies in pregnant women were found to be significantly higher than those induced by COVID-19 infection during pregnancy and the second dose of the vaccine was essential for pregnant women to achieve an adequate immune response [52]. Overall, the COVID-19 vaccination has established positive outcomes for pregnant individuals without major side effects, offering a crucial tool to protect both maternal health and the health of their fetus with lasting effects into infancy.

### 5.5. COVID-19 and Magnesium

Magnesium sulfate is a commonly administered medication in obstetrics for the effective prevention and control of premature labor, gestational hypertension, preeclampsia, and eclampsia [53]. Magnesium plays a significant role in producing an adequate immune response. Citu I.M. et al. found that expectant mothers who received a diet enriched with calcium, zinc, and magnesium, or magnesium alone, did not exhibit a distinct clinical difference during a COVID-19 illness. However, they did display a notably elevated level of anti-COVID-19 antibodies [54]. Given magnesium’s pivotal role in immune function and the advantageous impact of magnesium sulfate in averting and managing conditions such as premature labor, gestational hypertension, preeclampsia, eclampsia, and cerebral palsy, timely administration of MgSO4 should be recommended for pregnant women in the setting of COVID-19 infection [53].

## 6. Summary and Conclusions

The COVID-19 pandemic swept over the world at an alarming speed and changed the very foundation of our society. Several populations were at risk and required special attention secondary to the rapidly growing virus, including pregnant patients. Since the emergence of SARS-CoV-2, there have been an innumerable case series, case reports, reviews and scientific articles published regarding the effects that COVID-19 infection has on maternal health, fetal health and pregnancy in general. Mothers infected with SARS-CoV-2 had higher ventilation rates, lower rates of antenatal care, a high risk of obstetrical complications and an increased risk of being admitted to the ICU. COVID-19′s effect on the fetus was less marked with reports of mild elevations of fetal growth restrictions and some reports of increased prematurity. Overall, fetal complications reported were equivocal and modulated with the virulence of the virus. Direct attack by COVID-19 on the placenta, termed COVID placentitis, did occur but was extremely rare and has, to our knowledge, ceased to occur after the emergence of the less virulent Omicron variant of the virus. 

The practical implications of this review are demonstrating that women affected by COVID-19 did worse than their non-pregnant counterparts; complications of pregnancy such as preterm and preeclampsia were mildly increased in COVID-19; COVID-19 only rarely infected the placenta, causing COVID placentitis, which to our knowledge has not been reported since the Omicron era. COVID-19 did exert significant indirect adverse health influence by reducing access to care, including both antenatal visits and care in labor and delivery on a global scale. The mental health implications were also serious and have been somewhat understudied. This knowledge should inform healthcare decision making and broader public health policy as the emergence of new pandemics is a sad inevitability. 

Future perspectives include ongoing surveillance of the impact of endemic COVID-19 on pregnancy, long-term studies of the health outcomes of the cohort of people gestated and born during COVID-19 (this can be augmented by a DOHAD approach) and studies of the impact of Long COVID on maternal health and pregnancy. Additional future perspectives should include evaluation and development of care delivery systems that can ensure safe access to antenatal and intrapartum care during pandemic times.

The lessons to be learned from this global epidemic can be drawn from the teachings of the great writer Albert Camus in his book “The Plague”. Camus portrays a timeless reality of the human experience: our susceptibility to loss and pain, which ultimately affects every individual without exception. This should increase our sense of mutual responsibility, but it is a lesson so easily forgotten in good times, until the plague returns to teach us, again, about ourselves. COVID-19 has taught us about our deficits in maternal care. Women were unable to receive adequate maternal care, were contracting illness in labor and on delivery units of hospitals, and were being denied essential antenatal care. Pregnant patients have been disproportionately affected by these deficits, essentially causing greater harm than even the COVID-19 pandemic. 

## Data Availability

No new data were created or analyzed in this study. Data sharing is not applicable to this article.
